# SeaLSOD-YOLO: A Lightweight Framework for Maritime Small Object Detection Using YOLOv11

**DOI:** 10.3390/s26072017

**Published:** 2026-03-24

**Authors:** Jinjia Ruan, Jin He, Yao Tong

**Affiliations:** China Waterborne Transport Research Institute, Beijing 100088, Chinatongyao@wti.ac.cn (Y.T.)

**Keywords:** maritime target detection, small object, YOLO, feature fusion, dynamic sampling

## Abstract

Maritime small object detection is critical for UAV-based sea surveillance but remains challenging due to the small size of targets and interference from sea reflections and waves. This paper proposes SeaLSOD-YOLO, a lightweight detection algorithm based on YOLOv11, designed to improve small object detection accuracy while maintaining real-time performance. The method incorporates four key modules: Shallow Multi-scale Output Reconstruction, which fuses shallow and mid-level features to preserve fine-grained details; SPPF-FD, which combines spatial pyramid pooling with frequency-domain adaptive convolution to enhance sensitivity to high-frequency textures and suppress sea-surface interference; attention-based feature fusion, which emphasizes small object features through channel and spatial attention; and dynamic multi-scale sampling, which optimizes feature representation across different scales. Experiments on the SeaDroneSee dataset demonstrate that, compared with YOLOv11s, the proposed method improves precision from 75.6% to 81.9%, recall from 62.6% to 73.5%, and mAP@0.5 from 67.9% to 77.0%. The mAP@0.5:0.95 also increases from 41.1% to 44.9%. The model achieves an inference speed of 256 FPS. Although the parameter size increases from 18.2 MB to 30.8 MB, the method maintains a favorable balance between detection accuracy and computational efficiency. Comparative evaluation further shows superior performance in detecting small maritime objects such as buoys and lifeboats. These results indicate that SeaLSOD-YOLO effectively balances accuracy, efficiency, and real-time capability in complex maritime environments. Future work will focus on further optimization of attention mechanisms and upsampling strategies to enhance the detection of extremely small targets.

## 1. Introduction

Maritime small object detection is crucial for sea surveillance based on unmanned aerial vehicles (UAVs), enabling vessel monitoring, search and rescue, and environmental observation [1,2]. In marine environments—including ports, coastal areas, and ocean monitoring applications—UAVs are widely used for detection tasks [3], such as identifying structural cracks in infrastructure or ships for safety purposes. The aerial perspective introduces additional challenges, as targets appear smaller, backgrounds change dynamically, and variations in altitude and viewpoint exacerbate feature sparsity, increasing the difficulty of accurate detection. Many targets, such as buoys, life rafts, and small vessels, are small, densely distributed, and partially occluded [4]. Complex sea conditions, including illumination changes, sunlight glint, wave reflections, and adverse weather, further increase detection difficulty [5,6]. Therefore, accurately identifying tiny maritime objects is essential for operational safety and timely decision-making.

Early maritime detection methods relied on handcrafted features and traditional vision techniques [7,8,9,10,11], but they lacked robustness in complex sea conditions and required extensive manual tuning. With advances in deep learning, CNN-based detectors have become dominant [12,13,14]. Two-stage methods [15,16,17] achieve high localization accuracy but incur high computational costs, while single-stage detectors [18,19,20,21,22,23] like You Only Look Once (YOLO) provide faster inference suitable for UAV platforms. Researchers have introduced multi-scale fusion, attention mechanisms, and lightweight designs to improve small object performance [24,25,26,27]. However, maritime small object detection remains challenging. Deep features lack sufficient spatial detail, complex sea surfaces introduce significant background interference [28,29,30], and dense target distributions increase feature discrimination difficulty [31]. These challenges call for more effective shallow–deep feature integration and stronger background suppression strategies.

Consequently, there remains a pressing need for specialized network designs that preserve small object details, enhance feature representation across scales, and mitigate background interference. Addressing these challenges is crucial for achieving reliable detection of tiny, densely distributed maritime targets in real-world UAV imagery.

To address these challenges, we propose SeaLSOD-YOLO, short for Sea Lightweight Small Object Detection based on YOLOv11 [32]. The model emphasizes representational capability over aggressive parameter pruning, aiming to detect densely distributed small targets under complex sea-surface conditions with higher fidelity. A multi-scale enhancement pipeline is implemented to improve feature extraction for micro targets: shallow-layer multi-output recombination preserves fine spatial details from early layers while maintaining mid-to-high-level semantic information, ensuring that tiny targets, such as buoys and life rafts, retain discriminative features that are often lost in deep layers. Attention-guided feature fusion is applied to selectively highlight target-relevant features and suppress background noise from waves, reflections, or sunlight glint, improving the clarity of small object representations. Additionally, dynamic multi-scale sampling aligns features across different resolutions, allowing the model to maintain consistent representations for targets of varying sizes and ensuring that detection of extremely small objects, down to 16×16 pixels, is more reliable.

In addition to spatial enhancements, frequency-domain information is integrated through the Spatial Pyramid Pooling Fast module based on FDConv (SPPF-FD), which combines spatial pyramid pooling with frequency-domain adaptive convolution. This design amplifies high-frequency edges corresponding to small object textures while attenuating low-discriminative signals caused by environmental interference, such as wave patterns and sunlight reflections. By incorporating frequency-aware processing, the module strengthens the model’s sensitivity to subtle features of tiny maritime objects without substantially increasing computational cost. Together, the multi-scale recombination, attention-guided fusion, dynamic sampling, and frequency-domain enhancement form a cohesive framework that systematically improves the representation of small targets and mitigates background interference, providing a principled approach for UAV-based maritime small object detection under challenging real-world conditions.

The main contributions of this paper are as follows:

- We propose SeaLSOD-YOLO, a YOLOv11-derived framework that prioritizes representational power over parameter pruning to detect tiny, densely packed maritime objects amid sun glint and wave clutter.
- We design a multi-scale enhancement pipeline: shallow-layer multi-output recombination preserves fine spatial detail, attention-guided fusion suppresses background noise, and dynamic sampling aligns cross-scale features for 16×16 pixel targets.
- We embed frequency-domain adaptive convolution into the spatial pyramid pooling branch, yielding the SPPF-FD module that amplifies high-frequency edges of buoys and life rafts while dampening low-discriminative energy from sea glitter and reflection.
- Experimental results on the SeaDroneSee dataset demonstrate gains of 3.8% mAP@0.5 and 4.2% recall over the baseline, with consistent improvements on the most challenging small object categories, while preserving real-time inference performance for UAV-based maritime surveillance.

## 2. Related Work

### 2.1. Marine Target Detection

Early maritime detection methods relied on classical image processing and traditional machine learning. Shi et al. [33] combined background subtraction and edge detection for ship localization, while Haigang et al. [34] introduced visual attention with LBP features to refine regions of interest. Suzuki et al. [35] applied HOG features with an SVM classifier for small-vessel detection. Although computationally efficient, these approaches degraded under dynamic sea surfaces, scale variations, and low-contrast targets, prompting the transition to deep learning.

With the development of CNNs, researchers have widely adopted deep learning detectors in maritime scenarios [36]. Wei et al. [37] applied Faster R-CNN to the HRSC2016 dataset for ship detection. Bai et al. [38] improved YOLOv5 by adding a small object detection layer and Ghost modules, reducing parameters while increasing mAP. Wang et al. [39] proposed YOLO-StarLS with wavelet transforms and multi-scale feature extraction to enhance both accuracy and speed. Transformer architectures have also been introduced for maritime object detection: Qiu et al. [40] fused Vision Transformer (ViT) features with CNN representations to capture long-range dependencies, thereby improving robustness under complex sea conditions and occlusion. In SAR imagery, Wang et al. [41] and Xue et al. [42] employed YOLOv8-based models on SSDD and OpenSARShip datasets, achieving strong detection performance.

Despite these advances, small maritime targets remain challenging due to limited pixel coverage and weak feature representation, especially at long distances. These factors increase missed detections and misclassifications, motivating the development of more precise small object detection frameworks.

### 2.2. Small Object Detection

Small object detection (SOD) targets objects that occupy only a few pixels or a small proportion of the image [43]. Compared with general detection, SOD suffers more from spatial information loss and weak feature representation, leading to a persistent performance gap between small and large objects. Simply scaling existing detectors proves insufficient; effective solutions require dedicated architectural and feature-modeling designs.

Recent SOD research has focused on enhancing feature representation. Redmon et al. [44] promoted multi-scale modeling through feature pyramid structures, enabling the fusion of shallow spatial and deep semantic features. However, this fusion introduces a trade-off between localization precision and semantic richness. To address this issue, Lim et al. [45] incorporated attention mechanisms to emphasize target regions, while Xu et al. [46] and Cheng et al. [47] explored transformer-based and cross-attention designs to model long-range and cross-scale dependencies. Although these strategies improve robustness, excessive reliance on complex modules increases computational cost and may weaken fine-grained shallow features.

Beyond architecture design, Liu et al. [48] proposed size-aware loss functions and balanced label assignment to alleviate small object underrepresentation, while Bosquet et al. [49] enhanced training data through augmentation and synthetic generation. Some studies introduced super-resolution modules to recover high-frequency details, but improper designs risk introducing artifacts. These techniques generally serve as auxiliary enhancements rather than core solutions.

Despite these efforts, existing methods remain suboptimal in maritime scenarios characterized by dense small targets and repetitive backgrounds. Many approaches emphasize deep semantics or computationally intensive modules, which may hinder shallow feature preservation and real-time deployment. These limitations highlight the need for lightweight, shallow-feature-oriented SOD frameworks, motivating the method proposed in this work.

### 2.3. Feature Fusion

Feature fusion is essential in deep learning, allowing networks to combine low-level details with high-level semantics [50] to capture objects across scales and improve robustness to appearance changes, occlusion, and context variations.

Early methods like FPN [51] used top-down and lateral connections for multi-scale integration, while more recent approaches employ attention-based weighting and transformer modules to enhance informative features and long-range interactions [52]. Multi-branch [53] and multi-modal [54] fusion further combine features from parallel streams or different sensors, strengthening representation under occlusion, scale variation, and complex lighting, and consistently improving performance across domains.

Building on these advances, this work proposes a feature fusion module for maritime object detection, integrating adaptive attention with cross-layer interactions to enhance multi-scale representation and improve small object detection under challenging sea-surface conditions.

## 3. Methods

This section presents SeaLSOD-YOLO, including descriptions of the YOLOv11 and SeaLSOD-YOLO architectures, followed by the proposed improvements—Shallow Multi-scale Output Reconstruction, Feature Fusion, and SPPF-FD—and the loss function.

### 3.1. YOLOv11 Model

**Figure 1:**
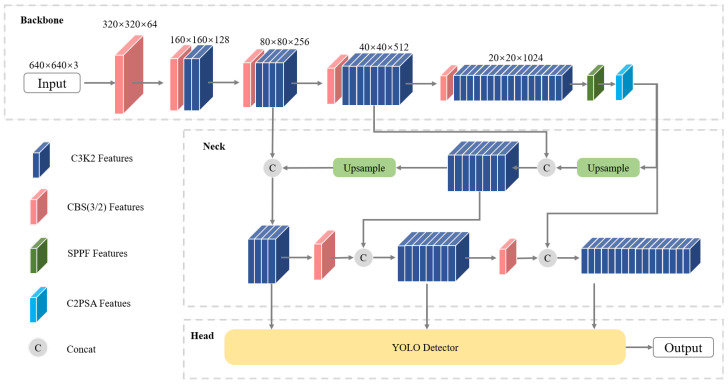
YOLOv11 model architecture.

As shown in Figure 1, YOLOv11 employs a modular detection architecture comprising a backbone, neck, and detection head. The backbone extracts hierarchical feature representations, the neck facilitates multi-scale feature fusion, and the detection head performs object classification and bounding box regression.

The architecture balances detection accuracy and computational efficiency through the C3K2 module for enhanced feature extraction, structural reorganization enabling deeper networks with a moderate parameter cost, the SPPF module for multi-scale context aggregation, and the C2PSA attention module to emphasize critical features. These components collectively support robust performance in general multi-scale object detection.

Nonetheless, maritime targets are typically small, densely distributed, and embedded in complex backgrounds, which can lead to detail loss and insufficient integration of shallow and deep features. Consequently, targeted modifications to the YOLOv11 architecture are required to address the specific challenges of small object detection in maritime scenarios.

### 3.2. SeaLSOD-YOLO

**Figure 2:**
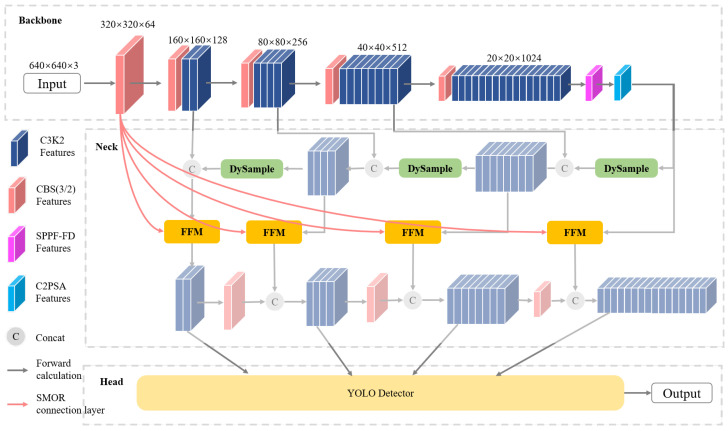
The SeaLSOD-YOLO model architecture.

This work proposes SeaLSOD-YOLO, a model based on the YOLOv11-s series that is specifically designed for maritime object detection. It improves small object detection accuracy while enhancing robustness against reflections and wave patterns on the water surface. The overall architecture of SeaLSOD-YOLO is illustrated in Figure 2 and, similar to YOLOv11, consists of a backbone, neck, and detection head.

To enhance small object detection, this work introduces Shallow Multi-scale Output Reconstruction (SMOR), which injects high-resolution shallow features into the neck outputs through cross-layer connections. This reconstruction strengthens the model’s feature representation for small objects and enables higher detection precision.

The model also incorporates DySample and feature fusion modules. DySample is a lightweight upsampling operator that replaces traditional nearest-neighbor interpolation, improving upsampling accuracy and preserving fine-grained details critical for small object detection. The feature fusion module, based on an attention mechanism, addresses asymmetry in the merging of shallow and deep features, enhancing the consistency of multi-scale feature integration.

Furthermore, SeaLSOD-YOLO introduces the SPPF-FD module. By integrating frequency dynamic convolution into the SPPF, this module adaptively modulates multi-scale context aggregation according to different frequency components, mitigating excessive smoothing of small object details in deep features.

### 3.3. Shallow Multi-Scale Output Reconstruction

**Figure 3:**
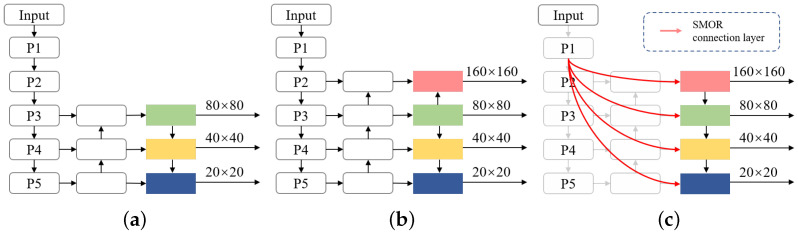
Structure of the LSOD-YOLO model. (**a**) YOLOv11 baseline structure. (**b**) YOLOv11 (with added P2 layer detection head). (**c**) Proposed SeaLSOD-YOLO model.

Conventional detection models increase network depth to enrich semantic representation, but repeated downsampling progressively weakens fine-grained spatial information, limiting the preservation of edges and structural details in small objects. In the YOLOv11 backbone, the input image passes through five downsampling stages, producing feature maps P1–P5 at different scales. As shown in Figure 3a, these maps trade off spatial resolution and semantic abstraction, three of which are selected for multi-scale prediction.

Deep feature maps have low spatial resolution, causing localization errors for small objects due to positional information loss during pooling and downsampling. Shallow feature maps retain higher resolution and richer edge cues, aiding accurate localization and shape modeling. Incorporating shallow features into multi-scale fusion is therefore crucial [51]. Some methods add the P2 layer to the neck and use an extra detection head to leverage higher-resolution features (Figure 3b), improving small object sensitivity. However, such designs limit interaction between shallow and deep features, restricting the influence of fine-grained information across semantic levels.

To address these limitations, this work proposes an SMOR mechanism. Considering that deep features primarily encode high-level semantic information, while shallow features are more sensitive to the edges and fine details of small objects, SMOR uses the P1 feature map output by the backbone, $F_{1}\in R^{C\times H\times W}$, as a high-resolution information source. After scale alignment via downsampling, $F_{1}$ is injected into multiple-scale branches $F_{i}\left(i=2,3,4,5\right)$ in the neck for feature fusion and reconstruction. The cross-layer fusion process can be formulated as follows:

(1) $$\hat{F_{i}}=f_{FFM}\left(Down\left(F_{1}\right),F_{i}^{\prime }\right),i=2,3,4,5$$

where Down(·) denotes the downsampling operation, and $f_{FFM}\left(\right)$ represents the Feature Fusion Module described in Section 3.4.2.

This cross-layer output reconstruction allows shallow fine-grained information to contribute across semantic levels, preserving consistency in deep features while reducing small object detail loss. As shown in Figure 3c, SMOR enhances collaboration between shallow and deep features, producing more discriminative multi-scale representations for the detection heads.

### 3.4. Feature Fusion of SeaLSOD-YOLO

In SeaLSOD-YOLO, the neck employs a PAN-FPN (Path Aggregation Network–Feature Pyramid Network) architecture to enable bidirectional multi-scale feature flow. Combined with SMOR, high-resolution shallow features from P1 are injected into the neck, integrating fine-grained spatial details with high-level semantic representations. To improve upsampling accuracy and cross-layer alignment, the neck incorporates dynamic sampling and a Feature Fusion Module, enhancing detail preservation and cross-scale consistency during feature reconstruction.

#### 3.4.1. DySample

YOLOv11 typically uses nearest-neighbor interpolation for upsampling, which ignores feature distribution and may distort small object details. DySample addresses this by dynamically generating sampling offsets to adapt the regular grid, creating content-aware sampling points on the feature map. Unlike convolution-based or subnetwork-based methods, DySample predicts offsets through linear mapping with pixel shuffle, improving local structure modeling while maintaining low computational cost [55].

**Figure 4:**
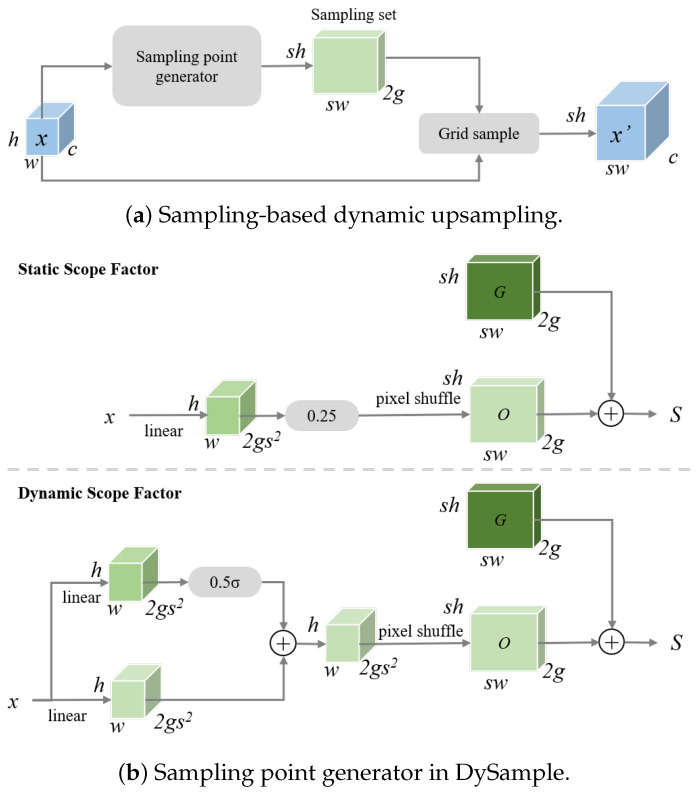
The network architecture of DySample.

Specifically, as illustrated in Figure 4, DySample starts from a regular sampling grid $G\in R^{sH\times sW\times 2g}$. Corresponding sampling offsets $O\in R^{sH\times sW\times 2g}$ are predicted through a linear layer to dynamically refine the sampling positions. The upsampling process can be formulated as follows:

(2) $$X^{\prime }=GridSample\left(X,S\right)$$

where *S* denotes the sampling set. $X\in R^{H\times W\times C}$ denotes the input feature map, $X^{\prime }\in R^{sH\times sW\times C}$ represents the upsampled high-resolution feature map, and sH and sW denote the height and width after upsampling, respectively.

DySample supports two offset-generation strategies controlled by scope factors. For the static scope factor, a fixed scaling factor α is applied to the linear mapping output:

(3) $$O_{static}=PixelShuffle\left(\alpha \cdot Linear(X)\right)$$

where α is typically set to 0.25 to constrain the offset magnitude within a stable range. The Linear(·) operation denotes a learnable linear mapping layer that projects the input features into a predicted-offset space. The PixelShuffle(·) operation rearranges the offset tensor from the channel dimension into the spatial dimensions to produce high-resolution offset fields.

For the dynamic scope factor, offsets are adaptively modulated by local feature responses:

(4) $$O_{dynamic}=PixelShuffle\left(f_{\mathrm{mul}}\left(\mathrm{Linear}\left(X\right),\alpha \cdot f_{Sigmoid}\left(\mathrm{Linear}(X)\right)\right)\right)$$

where α is set to 0.5.

The final sampling set *S* is obtained by combining the predicted offsets with the original grid:

(5) S=O+G

Based on the refined sampling positions, DySample generates an upsampled feature map of size C×sH×sW. This adaptive sampling mechanism enables more effective preservation of local structural details of small objects during feature reconstruction, thereby reducing feature distortion introduced by conventional upsampling methods.

#### 3.4.2. Feature Fusion Module

During multi-scale fusion in the neck, shallow features carry rich spatial details, while deep features encode global semantics. Direct element-wise addition can cause interference, reducing discriminative power.

**Figure 5:**
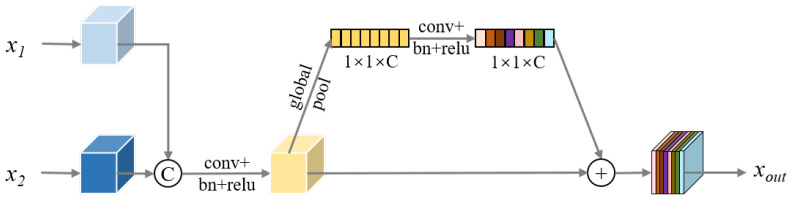
The feature fusion module architecture.

To address this, we propose a Feature Fusion Module (FFM), illustrated in Figure 5, for adaptive integration of shallow and deep features. Shallow features from P1 are concatenated with the corresponding deep features along the channel dimension and are then processed through convolution, normalization, and nonlinear activation to refine the representation.

FFM further applies a channel-attention mechanism inspired by SENet [56]. Fused features undergo global pooling and linear transformations to produce a channel-wise weight vector, which recalibrates feature channels, emphasizing task-relevant components and suppressing interference. This enables unified modeling of shallow and deep information, providing detection heads with more consistent and discriminative multi-scale representations.

### 3.5. SPPF-FD

SPPF-FD is an enhanced version of YOLOv11’s SPPF module, designed to improve fine-grained feature representation of small objects. While the original SPPF enlarges the receptive field via sequential pooling, repeated pooling smooths high-frequency details, limiting small object detection. SPPF-FD addresses this by incorporating frequency dynamic convolution (FDConv) [57] during channel fusion.

**Figure 6:**
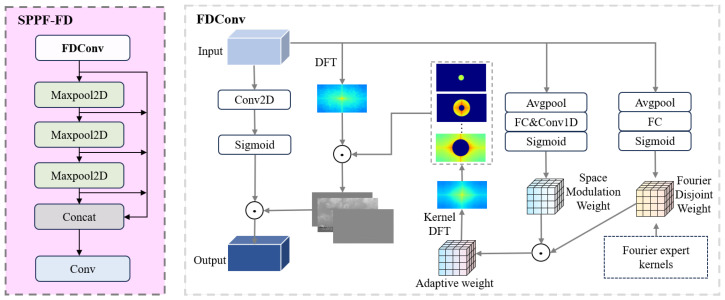
The SPPF-FD architecture.

As illustrated in Figure 6, FDConv generates convolution weights based on the spectral properties of input features. The input features are mapped to the frequency domain via the discrete Fourier transform to extract magnitude and phase information. Weight matrices are then dynamically generated to modulate convolution kernels, enhancing mid- to high-frequency details while preserving deep semantic information.

In SPPF-FD, FDConv replaces 1×1 convolutions in the original SPPF, both before and after pooling. Multi-scale pooled features are processed with FDConv in the frequency domain and then fused, maintaining spatial dimensions and the receptive field while introducing frequency-adaptive modulation. This approach improves retention of edges and structural details in small maritime targets and suppresses high-frequency noise from waves and reflections, yielding more robust features for subsequent fusion and detection.

### 3.6. Loss Function

During model training, we adopt the native composite loss function of YOLOv11, which integrates CIoU, BCE, and DFL losses to simultaneously optimize localization and classification performance. The CIoU loss improves bounding box precision by considering the center distance, aspect ratio, and overlap between predicted and ground-truth boxes. The BCE loss focuses on classification, minimizing the discrepancy between predicted and ground-truth class labels to achieve accurate object recognition. The DFL loss further enhances regression accuracy by modeling the distribution of bounding box predictions. The overall loss is computed as follows:

(6) $$Loss=\lambda_{1}L_{CIoU}+\lambda_{2}L_{cls}+\lambda_{3}L_{dfl}$$

where $L_{CIoU}$, $L_{cls}$, and $L_{dfl}$ denote CIoU, BCE, and DFL losses. $\lambda_{1}$, $\lambda_{2}$, and $\lambda_{3}$ are 7.5, 0.5, 1.5, respectively.

## 4. Dataset and Experimental Setup

### 4.1. Overview of the Experimental Dataset

To investigate the technical challenges associated with maritime small object detection, this study conducted experiments using the SeaDronesSee [58] dataset. This dataset is designed to support UAV-based maritime search and rescue scene perception tasks and was collected using multiple UAV platforms in real ocean environments, featuring high scene complexity and task-specific relevance. The SeaDronesSee dataset contains a total of 14,227 RGB images, of which 8930 are used for training, 1547 for validation, and 3750 for testing. The image acquisition heights range from 5 m to 260 m, and the UAV gimbal pitch angles span 0° to 90°, reflecting object distributions under varying scales and viewing perspectives. The dataset includes several object categories closely related to maritime SAR, such as swimmers, boats, and buoys.

To explore the challenges in maritime object detection, the objects in the SeaDronesSee dataset were divided into four size scales: tiny objects (smaller than 16 × 16 pixels), small objects (between 16 × 16 and 32 × 32 pixels), medium-sized objects (between 32 × 32 and 96 × 96 pixels), and large objects (larger than 96 × 96 pixels). The size distribution is summarized in Table 1. The “count” column in the table is used to tally the number of objects (excluding the count of images). For instance, 5688 indicates that there are 5688 boats with an area less than 16 × 16 in the dataset, and they account for 37.33% of all boats. Notably, objects smaller than 16 × 16 pixels account for 69.79% of the dataset. In the “swimmer” and “life_saving_appliances” categories, the proportions of small and tiny objects are 82.65% and 99.60%, respectively, highlighting the dataset’s class imbalance and the predominance of small objects, both of which increase detection difficulty. Furthermore, many targets in the SeaDronesSee dataset exhibit characteristics such as small scale, strong background interference, and large appearance variation, placing higher demands on the model’s feature representation and multi-scale modeling capabilities.

### 4.2. Experimental Platform

The training and evaluation were conducted on a Linux system. The GPU utilized was an NVIDIA GeForce RTX 5090, and the system was powered by CUDA version 12. Python 3.12 was used as the programming language and was managed through Conda version 24.9.2, and the core deep learning framework was PyTorch version 2.4.1.

Hyperparameters play a crucial role in our experiments. The input image size was set to 640 × 640, determining the resolution of training samples and influencing both detection performance and computational efficiency. The learning rate was initialized at 0.01, controlling the step size of weight updates, while the batch size was set to auto-batch to maximize GPU utilization adaptively. The number of epochs was set to 100 to ensure sufficient training for convergence. The optimizer selected was stochastic gradient descent (SGD). Standard data augmentations, including HSV color jitter, rotation, translation, scaling, and horizontal and vertical flipping, were applied throughout training to enhance model generalization.

### 4.3. Evaluation Metric

The evaluation indicators used in this study were grouped into three categories: accuracy indicators, computational cost indicators, and visual inspection of detection results. They followed the evaluation indicators of the literature [59].

The basic accuracy indicators included precision (P), recall (R), and average precision (AP). Precision represents the reliability of the model’s predictions and is calculated as the ratio of true positives (TP, namely, correctly detected objects) to the sum of true positives and false positives (FP, namely, false alarms). Recall reflects the model’s ability to detect all target objects and is calculated as the ratio of true positives to the sum of true positives and false negatives (FN, namely missed instances).

(7) $$\mathrm{Precision}=\frac{TP}{TP+FP}$$

(8) $$\mathrm{Recall}=\frac{TP}{TP+FN}$$

Average precision is obtained by computing the area under the precision–recall curve, providing a comprehensive measure of detection performance across different recall thresholds:

(9) $$AP=\int_{0}^{1}P\left(r\right)\;dr$$

For multi-class detection tasks, the mean average precision (mAP) is calculated by averaging the AP values across all object categories:

(10) $$mAP=\frac{1}{C}\sum_{i=1}^{C}AP_{i}$$

where *C* denotes the total number of categories. The extended indicators mAP50 and mAP50–95 employ fixed and dynamic IoU threshold strategies, respectively. mAP50 uses a fixed IoU of 0.5 as the localization accuracy benchmark, while mAP50–95 averages AP over IoU thresholds from 0.5 to 0.95 with a step size of 0.05, evaluating the model’s robustness to positional deviations of small objects.

Computational cost indicators include model size, GFLOPs, and inference time per image, which provide insights into the efficiency and deployability of the model.

Finally, detection results are visualized to qualitatively assess prediction correctness, bounding box placement, and confidence scores. This qualitative inspection complements quantitative metrics and helps verify the model’s practical performance in maritime small object scenarios.

## 5. Results and Discussion

### 5.1. Overall Performance Comparison Before and After Improvement

To evaluate the effectiveness and accuracy of the proposed method in maritime UAV reconnaissance scenarios, comparative experiments were conducted using YOLOv11s and its enhanced variant, YOLOv11s-P2, which incorporates an additional small object detection layer. All experiments were performed on the SeaDroneSee dataset under identical training settings and parameter configurations to ensure a fair comparison.

**Figure 7:**
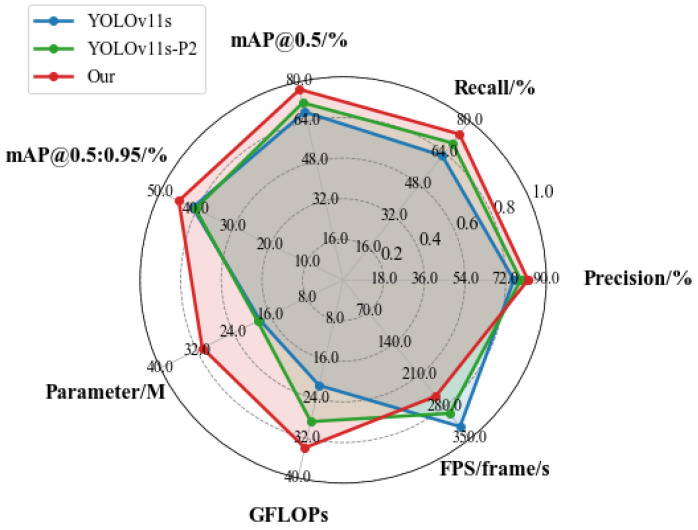
Radar comparison chart with each model.

The overall performance comparison is illustrated in the radar chart (see Figure 7), with detailed quantitative results reported in the corresponding tables. Compared with the original YOLOv11s, YOLOv11s-P2 achieves noticeable improvements in detection performance, with precision increasing from 75.64% to 78.66%, recall from 62.58% to 69.01%, and mAP@0.5 improving by 3.74 points. These results indicate that introducing an additional detection layer with higher-resolution features is beneficial for small object detection in maritime scenes. However, its mAP@0.5:0.95 slightly decreases from 41.1% to 40.39%, while the computational cost increases significantly, with GFLOPs rising from 21.3% to 28.6%, suggesting limited gains in high-IoU localization accuracy.

In contrast, the proposed method further improves detection accuracy across all evaluation metrics. Precision and recall reach 81.87% and 73.47%, respectively, while mAP@0.5 and mAP@0.5:0.95 increase to 76.97% and 44.92%, outperforming both YOLOv11s and YOLOv11s-P2. Although the model introduces additional parameters and computational overhead (30.8 MB and 33.9 GFLOPs), it achieves a real-time inference speed of 256 FPS on the experimental GPU platform.

The performance comparison by category further reveals the advantages of the proposed method, as shown in Table 2. For large-scale objects with prominent visual characteristics, such as boat and jetski, all three methods achieve high detection accuracy, and the proposed approach consistently yields the best mAP@0.5 and mAP@0.5:0.95 scores. For typical small object categories, including swimmer, buoy, and life_saving_appliances, the performance gap becomes more pronounced. Specifically, for the life_saving_appliances category, YOLOv11s achieves an mAP@0.5 of 28.02%, which improves to 34.63% with YOLOv11s-P2, while the proposed method further increases it to 45.86%. The corresponding mAP@0.5:0.95 also improves from 12.5% to 20.4%, indicating enhanced robustness in small object localization under complex maritime backgrounds.

**Figure 8:**
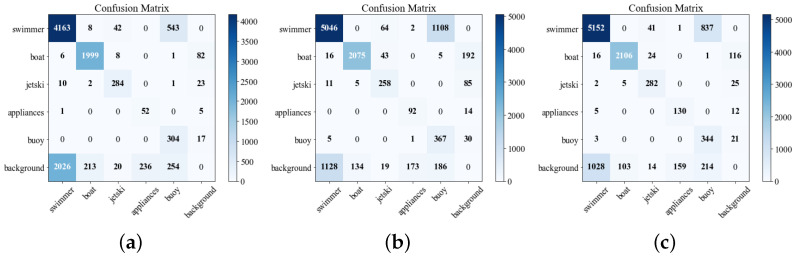
Confusion matrix of each model. (**a**) YOLOv11 baseline structure. (**b**) YOLOv11 (with added P2 layer detection head). (**c**) Proposed SeaLSOD-YOLO model. Note: appliances mean life_saving_appliances.

The confusion matrix analysis provides further insight into the classification behavior of different methods, as shown in Figure 8. The original YOLOv11s exhibits substantial confusion between a small object category, such as swimmer, and the background class. While YOLOv11s-P2 reduces this confusion to some extent, the proposed method shows a more concentrated diagonal distribution for key categories, including swimmer, jetski, and boat, with significantly reduced misclassification as the background. This result confirms the effectiveness of the proposed approach in mitigating false negatives and false positives for small objects in maritime UAV scenarios.

### 5.2. Comparison with State-of-the-Art Methods

To comprehensively evaluate the detection performance of the proposed SeaLSOD-YOLO framework, we systematically compared it with representative detectors, including Faster R-CNN and SSD, conventional single-stage YOLO variants (YOLOv3, YOLOv3-Tiny, YOLOv5, YOLOv6–v12), the real-time transformer-based detector RT-DETR, and several recent models (Enhanced YOLO11 [60], SAQ-YOLO [61], and MFEF-YOLO [62]). The experimental results are summarized in Table 3 and Table 4.

As shown in Table 3, Faster R-CNN and SSD achieve moderate precision (72.3% and 71.9%) but relatively low recall (48.5% and 52.9%), leading to mAP@0.5 scores of 53.3% and 50.8%, respectively. This indicates that two-stage detectors are limited in detecting small maritime objects, likely due to coarse region proposals and insufficient high-resolution feature representation. YOLOv3 and its variants (YOLOv5s, YOLOv6s–v12s) improve recall significantly (up to 63.8% for YOLOv3), benefiting from dense predictions and multi-scale feature aggregation, yet their mAP@0.5:0.95 values remain below 42.0%, highlighting challenges in the robust detection of small objects under complex sea-surface backgrounds. Lightweight models such as YOLOv3-Tiny and YOLOv5s achieve high FPS (up to 476) with low parameter counts, making them suitable for scenarios requiring high real-time performance; however, their mAP@0.5 scores (45.9% and 66.7%) reveal limited detection accuracy, particularly for small targets.

Recent optimized variants, including Enhanced YOLO11, SAQ-YOLO, and MFEF-YOLO, demonstrate notable improvements in precision for small object detection. Enhanced YOLO11 achieves the highest performance among these with 81.3% precision, 69.4% recall, 74.6% mAP@0.5, and 42.7% mAP@0.5:0.95. SAQ-YOLO attains comparable precision (81.2%) but suffers from lower recall (64.8%), resulting in inferior mAP@0.5 (71.3%) and mAP@0.5:0.95 (41.2%), suggesting that its spatial attention query mechanism enhances classification accuracy yet struggles with complete small object retrieval. MFEF-YOLO, employing multi-scale feature enhancement, achieves 80.9% precision and 67.7% recall with 73.6% mAP@0.5, offering a better balance between precision and recall than SAQ-YOLO but still falling short of Enhanced YOLO11. Nevertheless, all three methods fail to exceed 43.0% in mAP@0.5:0.95 and exhibit substantial recall gaps (up to 8.7%) compared to SeaLSOD-YOLO, indicating that existing improvement strategies still face challenges in jointly optimizing high recall and precise localization for small maritime objects under complex sea-surface conditions. Furthermore, none of these methods reports computational complexity or inference speed, leaving their real-time deployment capabilities uncertain.

RT-DETR and YOLOv11s achieve competitive accuracy, with mAP@0.5 of 68.0% and 67.9%, respectively. Nevertheless, RT-DETR has relatively high computational complexity (108 GFLOPs) and lower FPS (175), constraining its real-time applicability. In contrast, SeaLSOD-YOLO attains the highest overall performance, with a precision of 81.9% and recall of 73.5%, yielding mAP@0.5 of 77.0% and mAP@0.5:0.95 of 44.9%. While its parameter count (30.8 MB) and GFLOPs (33.9) are slightly higher than those of some lightweight YOLO variants, SeaLSOD-YOLO maintains real-time inference at 256 FPS, achieving an optimal trade-off between accuracy, speed, and model size. This work emphasizes balancing detection accuracy and computational efficiency rather than merely minimizing model size. Although additional lightweight optimizations, such as half-precision inference, have not yet been incorporated, a further streamlined YOLO-small variant is expected to satisfy real-time deployment requirements on mid-range UAV platforms (e.g., RK3588). Considering that maritime emergency rescue scenarios demand high detection reliability, the current trade-off between accuracy and efficiency is both reasonable and practically justified.

The superior performance of SeaLSOD-YOLO can be attributed to the integration of specialized modules for small maritime object detection. The SMOR module enhances shallow multi-scale feature representation, preserving fine-grained details of small targets. The SPPF-FD module improves sensitivity to high-frequency object textures while suppressing wave and reflection interference. Attention-guided feature fusion selectively emphasizes salient features from shallow and deep layers, and dynamic multi-scale sampling further refines object representation across scales. Together, these modules improve detection robustness and accuracy for small and densely distributed maritime targets.

Overall, the results demonstrate that SeaLSOD-YOLO not only outperforms existing state-of-the-art detectors in small object detection under challenging maritime conditions but also maintains computational efficiency suitable for real-time UAV deployment. These results confirm its effectiveness in balancing detection accuracy, real-time capability, and resource efficiency.

### 5.3. Component-Wise Performance Analysis

#### 5.3.1. Evaluation of Multi-Scale Connection Variants

To understand the effect of multi-scale feature connections on detecting objects of varying sizes, we systematically analyzed different shallow-to-deep layer connections in the SeaLSOD-YOLO architecture, as shown in Table 5. Specifically, we evaluated the removal of the P1 connections to N2, N3, N4, and N5 individually, which represent the integration of the shallowest layer with progressively deeper layers.

Removing the P1-N2 connection results in a small decline in total mAP to 76.5%, with the most noticeable decrease in tiny objects (from 76.1% to 75.4%). This indicates that the P1-N2 path is particularly important for preserving high-resolution features required for detecting the smallest targets, such as buoys and lifesaving appliances, which are sensitive to spatial detail. Excluding P1-N3 causes the total mAP to drop to 75.5%, suggesting that mid-level semantic information combined with shallow details is critical for balancing small and medium object detection. The absence of P1-N4 slightly reduces the total mAP to 76.3%, reflecting that this connection helps propagate context-aware features without overwhelming the fine-grained details. Notably, removing the P1-N5 connection produces the largest decline in overall mAP to 74.7%, with a significant drop for large objects (from 44.9% to 39.3%). This indicates that direct interaction between the shallowest and deepest layers strengthens high-level semantic representations, which are essential for accurately localizing large objects under complex maritime backgrounds.

The full connection setup, which retains all P1-to-N2–N5 links, achieves the highest total mAP of 77.0%, demonstrating that comprehensive multi-scale integration not only preserves fine-grained details for tiny objects but also reinforces semantic consistency for medium and large targets. The ablation results confirm that each P1 connection contributes differently: lower-level connections (N2, N3) mainly affect tiny and small objects, while higher-level connections (N4, N5) improve medium and large object representation, showing a complementary hierarchy of multi-scale feature fusion.

In addition, we observe that removing P1-N2, P1-N3, or P1-N4 unexpectedly improves mAP for large objects, which we attribute to the extremely low proportion of large objects (only 0.59%), making this metric highly susceptible to fluctuations, combined with the interference of low-level connections with gradient backpropagation to higher layers. Therefore, we argue that performance on tiny and small objects is more critical for maritime lifesaving small object detection, and the Full Connection design achieves optimal total mAP (77.0%) by preserving complete low-level feature pathways. We recommend future evaluation on balanced datasets or adopting weighted connection fusion strategies to further improve large object handling.

#### 5.3.2. Ablation Study

To quantify the individual contributions of key modules in SeaLSOD-YOLO, we conducted ablation experiments on DySample, the Feature Fusion Module (FFM), SMOR, and SPPF-FD, as summarized in Table 6.

Removing the DySample module slightly decreases the total mAP to 76.1%, with a modest reduction in medium and large object detection, suggesting that dynamic multi-scale sampling mainly benefits accurate representation across scales. Without FFM, total mAP remains comparable at 76.2%, but the performance for large objects drops sharply to 39.9%, highlighting that attention-guided fusion of shallow and deep features is critical for mitigating background interference and enhancing large object localization in complex maritime scenes. The joint removal of DySample and FFM reduces large object detection further to 37.3%, confirming that these modules work synergistically to maintain robust multi-scale feature representation.

Excluding SMOR leads to an overall mAP of 75.5%, primarily affecting tiny objects (‘Tiny mAP’ drops from 76.1% to 74.5%). This validates that SMOR is essential for capturing fine-grained details of small maritime targets, which are easily missed due to low resolution and background clutter. Similarly, removing SPPF-FD reduces the total mAP to 75.8%, with a pronounced decrease in large object detection (36.4%), indicating that frequency-domain enhanced pooling effectively suppresses high-frequency noise, such as wave reflections, and reinforces texture-sensitive feature extraction. The combined removal of SMOR and SPPF-FD produces the lowest total mAP (74.9%), showing that these two modules complement each other by simultaneously enhancing low-level details and high-frequency feature robustness.

The full SeaLSOD-YOLO model, incorporating all modules and complete multi-scale connections, achieves the highest total mAP of 77.0%, with balanced performance across all object scales: tiny (76.1%), small (95.9%), medium (92.4%), and large (44.9%). These results confirm that each component—SMOR, SPPF-FD, FFM, and DySample—provides a specific functional benefit, and their integration produces synergistic effects that significantly enhance detection performance, particularly for small and densely distributed maritime objects under complex backgrounds. The detailed analysis of each component demonstrates that careful multi-scale design and module optimization are key to achieving high accuracy while maintaining computational efficiency suitable for real-time UAV deployment.

Table 7 presents the computational cost of ablating key components from our full model. Removing DySample incurs a negligible reduction (0.1 MB, 0.0 GFLOPs), indicating its lightweight design. FFM and SMOR contribute modestly to parameters (1.8 and 1.9 MB) and computation (0.4 and 0.5 GFLOPs). Notably, SPPF-FD dominates model complexity, accounting for 29.6% of parameters and 13.6% of FLOPs, underscoring its heavy reliance on dynamic frequency-domain convolutions.

**Figure 9:**
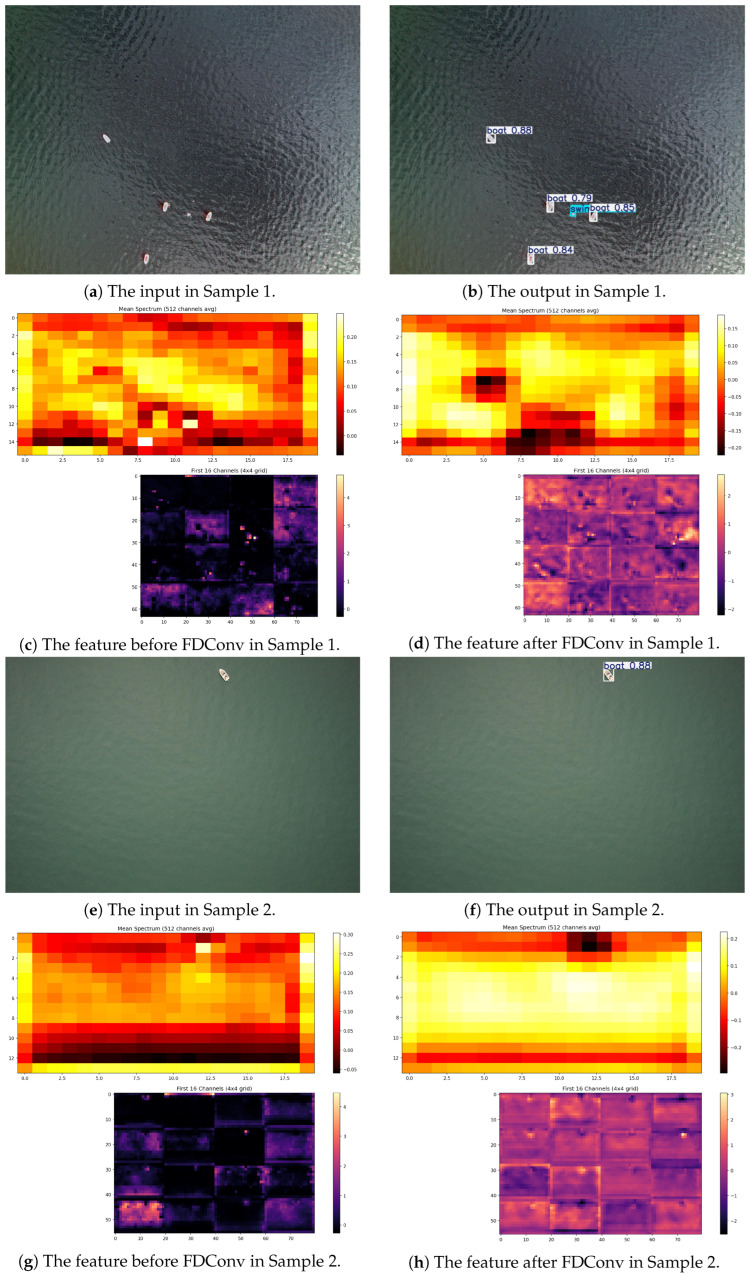
Feature map spectrogram before and after FDConv module.

In addition, we plotted the frequency spectra of the feature maps before and after the FDConv operator, as shown in Figure 9. And this demonstrates the frequency-domain feature enhancement mechanism of FDConv through spectral and spatial visualization of two representative samples. The average amplitude spectra reveal that FDConv processing substantially enhances energy distribution across multiple frequency bands (manifested as brighter spectral maps), indicating that the Fourier Disjoint Weight (FDW) mechanism successfully activates diverse frequency responses—where low-frequency components suppress noise and high-frequency components capture fine details simultaneously. Concurrently, the first 16 feature channels exhibit a marked transformation from sparse, inactive states to densely activated patterns with uniform brightness, confirming that FDConv effectively stimulates channel-wise feature propagation and enriches spatial representations. These observations validate that FDConv achieves frequency-space collaborative adaptation.

### 5.4. Visualization Analysis

The SeaDroneSee dataset employed in this study is specifically designed for open-water maritime search and rescue scenarios. It was collected using three UAV platforms, including two quadcopters from the DJI Mavic series and one fixed-wing drone. The flight altitudes range from 5 m to 260 m, covering typical maritime search and rescue conditions from low-altitude coastal surveillance to wide-area high-altitude monitoring. Therefore, this work evaluates SeaLSOD-YOLO from multiple UAV perspectives and diverse maritime scenes. Although the height and perspective metadata were not explicitly annotated for quantitative analysis, the detection results of SeaLSOD-YOLO can be demonstrated across multiple drone perspectives and maritime scenarios.

#### 5.4.1. Visual Analysis Under Different UAV Perspectives

**Figure 10:**
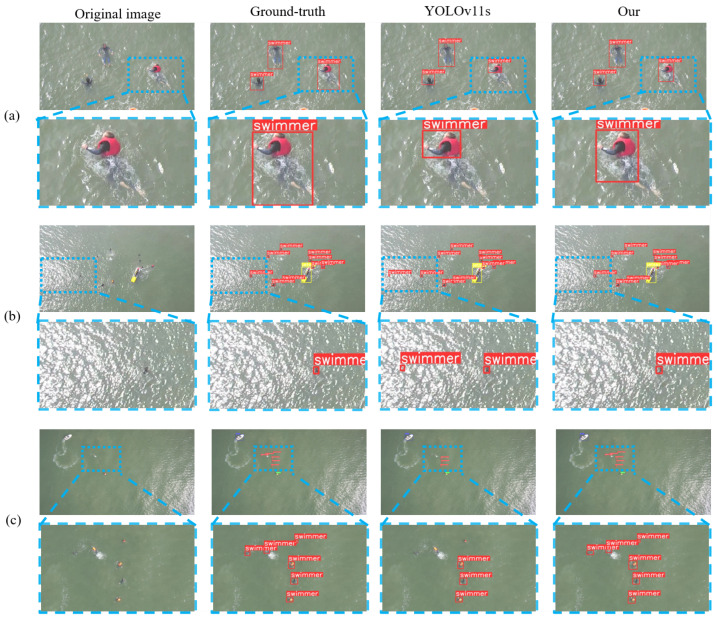
Comparison of maritime image detection results in different UAV perspectives. (**a**) near-vertical view at close range, (**b**) oblique view, and (**c**) near-vertical view from long range.

This work evaluates SeaLSOD-YOLO under multiple UAV perspectives using Figure 10, which presents three representative samples, each including the original image, ground truth (GT), YOLOv11s predictions, and SeaLSOD-YOLO predictions.

In sample (a), a near-range UAV view captures a swimmer partially submerged. GT marks the full-body bounding box. YOLOv11s detects only the exposed portion above the water, missing subtle cues from the submerged body. SeaLSOD-YOLO extends the predicted bounding box to cover part of the swimmer below the surface. This result demonstrates that shallow multi-scale feature fusion and attention-guided modules enhance the representation of partially occluded targets, allowing the network to leverage subtle visual textures underwater.

Sample (b) shows a distant oblique view containing several small and micro targets. YOLOv11s correctly identifies most swimmers but misclassifies a wave splash as a swimmer, likely because high-frequency reflections resemble swimmer textures. SeaLSOD-YOLO identifies all swimmers accurately and avoids false positives. The frequency-domain enhanced pooling and feature fusion modules enable the network to suppress wave-induced noise while retaining small object features, improving detection reliability under reflective water surfaces.

Sample (c) depicts another distant oblique perspective with a dense distribution of micro swimmers. YOLOv11s detects only three swimmers, missing half of the targets. SeaLSOD-YOLO detects all six swimmers, including the smallest visible instances. The SMOR preserves fine-grained details, while dynamic multi-scale sampling adapts to varied object scales. Together, these modules maintain high recall in scenarios with densely packed micro targets.

Across these UAV perspectives, SeaLSOD-YOLO consistently detects small, partially occluded, and densely distributed swimmers while suppressing false positives caused by water reflections and wave patterns. The results confirm that the proposed architecture effectively integrates shallow and deep features, enhances texture-sensitive representations, and maintains robustness under challenging maritime observation conditions.

#### 5.4.2. Visual Comparison Under Different Maritime Scenes

**Figure 11:**
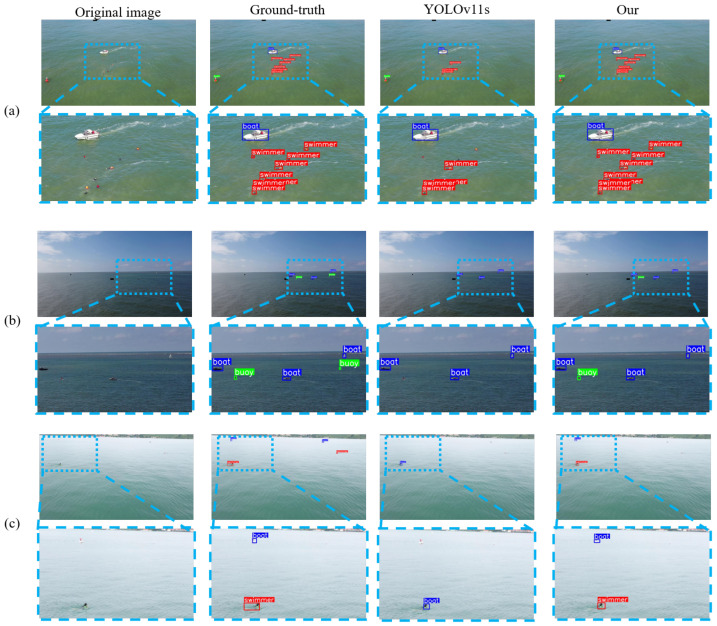
Comparison of maritime image detection results in different scenarios. (**a**) nearshore yellowish seawater with wave breaking, (**b**) deep ocean blue water, and (**c**) sunlit light cyan seawater.

This work evaluates SeaLSOD-YOLO under diverse maritime scenes (see Figure 11), which shows three representative samples with the original image, ground truth (GT), YOLOv11s predictions, and SeaLSOD-YOLO predictions.

Sample (a) depicts a nearshore scene with turbid yellowish water from the sea, containing numerous micro swimmers. SeaLSOD-YOLO successfully detects all seven swimmers, while YOLOv11s misses three. This demonstrates that the proposed SMOR and attention-guided feature fusion enable the network to capture subtle cues of micro targets in visually cluttered water.

Sample (b) shows a scene with clear cyan water and a distinct sea-surface boundary, containing micro targets near the water surface. Both methods miss some targets; however, SeaLSOD-YOLO correctly identifies a left-side buoy that YOLOv11s completely omits. This highlights the ability of frequency-domain enhanced pooling and multi-scale feature fusion to distinguish small, low-contrast objects against relatively uniform backgrounds, reducing omission errors for partially visible targets.

Sample (c) captures a nearshore cyan water scene with two swimmers and two boats, all appearing as micro targets. SeaLSOD-YOLO correctly detects one swimmer and one boat, while YOLOv11s detects only a single swimmer and misclassifies it as a boat. These results emphasize that SeaLSOD-YOLO maintains higher precision in multi-class micro-target detection, effectively handling scale ambiguity and preventing cross-class misclassification.

Across these diverse maritime scenarios, SeaLSOD-YOLO consistently outperforms YOLOv11s in detecting micro swimmers and small vessels, reducing both omission and misclassification rates. The combination of shallow and deep feature integration, attention-guided fusion, and frequency-domain processing enhances robustness to water turbidity, surface reflections, and low-contrast backgrounds, supporting reliable real-time maritime monitoring.

#### 5.4.3. Some Failures

**Figure 12:**
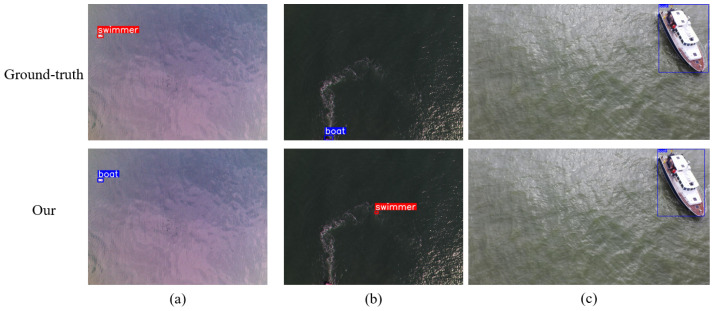
Some failures. (**a**–**c**) are respectively three failed samples selected from the dataset.

Despite the overall superior performance of SeaLSOD-YOLO in maritime micro-target detection, certain challenging scenarios still expose limitations in the proposed method. Figure 12 presents three representative failure cases that highlight specific weaknesses in detection robustness under complex maritime conditions. Sample (a) shows a tiny swimmer under strong specular reflection that is misclassified as a boat. Sample (b) depicts a partially occluded boat in heavy wave clutter that remains completely undetected, while some splashing waves are falsely recognized as swimmers. Sample (c) demonstrates a large-scale target with noticeable bounding box drift in our method’s prediction. These failures collectively underscore limitations in handling specular reflections, occlusion robustness, and scale generalization, motivating future research on explicit occlusion reasoning and multi-scale enhancement to achieve comprehensive maritime surveillance across diverse target sizes and environmental conditions.

## 6. Conclusions

This paper presents SeaLSOD, a lightweight framework for maritime small object detection based on YOLOv11. The framework addresses the critical challenges of detecting small and densely distributed targets, such as swimmers, buoys, and small boats, in complex maritime environments with varying water turbidity, reflections, and occlusions. We introduce several key modules to enhance detection performance while maintaining real-time efficiency. The SMOR module integrates shallow and mid-level features, preserving fine-grained details crucial for recognizing small targets. The SPPF-FD module incorporates frequency-domain adaptive convolution to suppress wave-induced noise and reflections while retaining high-frequency target textures. Attention-guided feature fusion emphasizes salient features and suppresses background interference, and dynamic multi-scale sampling adaptively adjusts feature resolution to accommodate targets of different scales.

We conducted extensive experiments on the SeaDroneSee dataset, including comparisons with state-of-the-art detectors such as YOLOv3–v12, RT-DETR, Faster R-CNN, and SSD. Both quantitative results and visual analyses demonstrate that SeaLSOD consistently achieves higher precision, recall, and mAP, particularly for micro and partially occluded targets. Ablation studies confirm that each module contributes meaningfully to the overall performance and that their integration produces a synergistic effect, balancing accuracy, real-time inference, and model efficiency. The visual evaluation under different UAV perspectives and maritime scenes further illustrates the robustness of SeaLSOD against challenging conditions such as turbid water, wave reflections, low-contrast backgrounds, and dense object distributions.

Beyond methodological improvements, the proposed framework has practical implications for maritime safety assurance, coastal monitoring, and emergency rescue operations, where reliable small object detection under real-time constraints directly contributes to operational efficiency and risk reduction. Potential future work includes extending the framework to handle multi-class maritime traffic scenarios, integrating temporal information from UAV video sequences to enhance detection stability, and exploring semi-supervised or self-supervised learning strategies to further improve performance under diverse environmental conditions.

## Figures and Tables

**Table 1:** The distribution of object size in the SeaDroneSee dataset.

Categories	Tiny		Small		Medium		Large	
	Count	Proportion	Count	Proportion	Count	Proportion	Count	Proportion
boat	5688	37.33%	5078	33.33%	4164	27.33%	306	2.01%
swimmer	35,788	82.65%	6556	15.14%	895	2.07%	63	0.15%
buoy	3063	61.89%	1759	35.54%	127	2.57%	0	0.00%
jetski	1243	46.91%	903	34.08%	474	17.89%	30	1.13%
life_saving_appliances	1248	99.60%	4	0.32%	1	0.08%	0	0.00%
All	47,030	69.79%	14,300	21.22%	5661	8.40%	399	0.59%

**Table 2:** Performance analysis across categories before and after the improvements.

Method	Category	mAP@0.5	mAP@0.5:0.95
YOLOv11s	swimmer	69.98	29.2
	boat	92.87	69.5
	jetski	88.1	56.6
	life_saving_appliances	28.02	12.5
	buoy	60.24	37.7
	Total	67.85	41.1
YOLOv11s-P2	swimmer	73.97	30.63
	boat	94.19	65.15
	jetski	81.85	49.65
	life_saving_appliances	34.63	15.53
	buoy	73.34	40.96
	Total	71.6	40.38
SeaLSOD-YOLO	swimmer	77.62	32.12
	boat	95.71	71.4
	jetski	88.39	55.2
	life_saving_appliances	45.86	20.4
	buoy	77.25	45.5
	Total	76.97	44.9

**Table 3:** Comparison of accuracy with state-of-the-art methods.

Method	Precision/%	Recall/%	mAP@0.5	mAP@0.5:0.95
Faster RCNN	72.3	48.5	53.3	31.7
SSD	71.9	52.9	50.8	28.2
YOLOv3	76.4	63.8	68.5	41.8
YOLOv3-Tiny	55.3	32.3	45.9	26.9
YOLOv5s	75.1	61.5	66.7	40.2
YOLOv6s	72.0	57.0	61.2	36.7
YOLOv8s	75.3	61.9	67.1	40.0
YOLOv9s	74.6	60.9	65.6	40.5
YOLOv10s	73.9	60.8	66.0	40.1
YOLOv11s	75.6	62.6	67.9	41.1
YOLOv12s	75.2	61.6	66.4	40.9
RT-DETR	76.0	62.9	68.0	42.0
Enhanced YOLO11	81.3	69.4	74.6	42.7
SAQ-YOLO	81.2	64.8	71.3	41.2
MFEF-YOLO	80.9	67.7	73.6	41.8
SeaLSOD-YOLO	81.9	73.5	77.0	44.9

**Table 4:** Comparison of computational costs with state-of-the-art methods.

Method	Param/MB	GFLOPs	FPS
Faster RCNN	43.1	223.7	128
SSD	25.4	88.1	222
YOLOv3	198.1	283.8	188
YOLOv3-Tiny	23.2	18.9	357
YOLOv5s	17.6	24.1	344
YOLOv6s	31.3	43.9	345
YOLOv8s	21.4	28.8	476
YOLOv9s	14.5	27.4	277
YOLOv10s	15.7	24.8	499
YOLOv11s	18.2	21.3	323
YOLOv12s	18.0	21.5	357
RT-DETR	32.2	108.0	175
Enhanced YOLO11	11.6	14.1	322
SAQ-YOLO	12.3	12.7	285
MFEF-YOLO	8.92	11.7	312
SeaLSOD-YOLO	30.8	33.9	256

**Table 5:** Ablation study on different connection layers.

Model	Tiny	Small	Medium	Large	Total
w/o P1-N2	75.4 (−0.7)	95.1 (−0.8)	91.1 (−1.3)	50.2 (+5.3)	76.5 (−0.5)
w/o P1-N3	74.5 (−1.6)	95.6 (−0.3)	91.3 (−1.1)	49.4 (+4.5)	75.5 (−1.5)
w/o P1-N4	75.3 (−0.8)	94.1 (−1.8)	93.3 (+0.9)	47.9 (+3.0)	76.3 (−0.7)
w/o P1-N5	73.4 (−2.7)	95.9 (−0.0)	93.2 (−0.8)	39.3 (−5.6)	74.7 (−2.3)
Full Connection	76.1	95.9	92.4	44.9	77.0

**Table 6:** Accuracy analysis of different model components.

Model	Tiny	Small	Medium	Large	Total
w/o DySample	74.9	95.4	90.6	51.5	76.1
w/o FFM	75.2	95.0	91.4	39.9	76.2
w/o DySample, w/o FFM	75.0	95.0	91.2	37.3	76.1
w/o SMOR	74.5	95.2	92.9	45.7	75.5
w/o SPPF-FD	75.1	95.8	92.5	36.4	75.8
w/o SMOR, w/o SPPF-FD	73.8	95.4	92.4	33.9	74.9
Full body	76.1	95.9	92.4	44.9	77.0

**Table 7:** The computational costs of different model components.

Model	Param/MB	GFLOPs
w/o DySample	30.7 (−0.1)	33.9 (−0.0)
w/o FFM	29.0 (−1.8)	33.5 (−0.4)
w/o SMOR	28.9 (−1.9)	33.3 (−0.5)
w/o SPPF-FD	21.7 (−9.1)	29.3 (−4.6)
Full body	30.8	33.9

## Data Availability

This work uses the open source database SeaDroneSee; the data can be obtained at https://seadronessee.cs.uni-tuebingen.de/ (accessed on 21 March 2026).
